# Complex network analysis of volatility spillovers between global financial indicators and G20 stock markets

**DOI:** 10.1007/s00181-022-02290-w

**Published:** 2022-09-10

**Authors:** Burak Korkusuz, David G. McMillan, Dimos Kambouroudis

**Affiliations:** Division of Accounting and Finance, University of Stirling, Stirling, FK9 4LA UK

**Keywords:** Volatility spillover, GARCH-BEKK, Complex network theory, Global financial indicators, G20 stock markets, C22, G12

## Abstract

This paper analyses the dynamic transmission mechanism of volatility spillovers between key global financial indicators and G20 stock markets. To examine volatility spillover relations, we combine a bivariate GARCH-BEKK model with complex network theory. Specifically, we construct a volatility network of international financial markets utilising the spatial connectedness of spillovers (consisting of nodes and edges). The findings show that spillover relations between global variables and G20 markets vary significantly across five identified sub-periods. Notably, networks are much denser in crisis periods compared to non-crisis periods. In comparing two crisis periods, Global Financial Crisis (2008) and COVID-19 Crisis (2020) periods, the network statistics suggest that volatility spillovers in the latter period are more transitive and intense than the former. This suggests that financial volatility spreads more rapidly and directly through key financial indicators to the G20 stock markets. For example, oil and bonds are the largest volatility senders, while the markets of Saudi Arabia, Russia, South Africa, and Brazil are the main volatility receivers. In the former crisis, the source of financial volatility concentrates primarily in the USA, Australia, Canada, and Saudi Arabia, which are the largest volatility senders and receivers. China emerges as generally the least sensitive market to external volatility.

## Introduction

A key concern for investors in seeking to build diversified portfolios is fluctuations in the relations between assets. Furthermore, that fluctuations in a market do not arise solely from internal information but are also affected by external information. The transmission of this external information across markets is known as ‘volatility spillover effects’ (see, for example, Yu et al. 2015; Rejeb and Arfaoui 2016; Mensi et al. 2018). This phenomenon is also referred to as fear connectedness by Diebold and Yilmaz (2014).

In the last two decades, the Global Financial Crisis (GFC, 2008) and the COVID-19 Crisis (CVC, 2020) evidence the importance and impact of spillover effects across financial markets. Here, the transmission of volatility across markets is likely to have a profound impact on each economy, varying with the degree of market integration. In the case of such crises, increasing globalisation and financialization of markets allows adverse effects in one market to further intensify existing spillover effects. Consequently, during such crises, investors typically sell-off risky assets on fears of financial contagion that results in a further spread of global risk. Therefore, there is a need to understand and model time-varying spillovers among a range of markets and how this could impact future investment behaviour.

In the spillover literature, there is a lack of empirical research that provides international evidence conducted on a large scale. The existing literature typically concentrates on spillovers between a small number of stock markets, often classified according to their level of development (e.g. emerging or developed) or considers a single stock market with several other assets (see, among others, Zhang et al. 2020a, b; Golosnoy et al. 2015; Piljak and Swinkels 2017; Yoon et al. 2019; Zhang et al. 2020a, b). As a larger dataset, the G20 stock markets may be thought of as a convenient research object to study volatility spillover effects as they account for over 85% of Gross World Product and over 80% of world trade. Thus, this bloc captures large changes to the world economy (Liu et al. 2017; Zhang et al. 2020a, b).

While a range of spillover models exist (e.g. stochastic volatility, Diebold and Yilmaz (2009, 2012) spillover index), an advantage of the GARCH-BEKK model is the ability to capture spillover effects as volatility can be directly computed from the variance–covariance matrix, without imposing any restriction on the conditional correlation structure (Lee et al. 2014). However, when examining the interrelations of multiple series, such as the G20, the GARCH-BEKK is not able handle the high multi-dimensional spillovers of the system as a whole. Therefore, a small number of studies (Liu et al. 2017; An et al. 2020; Zhang et al. 2020a, b) combine the econometric model with complex network theory to construct a network of financial markets. The advantage of this combined approach is to provide a solution to the difficulty encountered by the GARCH-BEKK model when dealing with multi-dimensionality and to provide a visualisation of the complex financial system in a clear way.

In the most relevant literature, Liu et al. (2017) combine the GARCH-BEKK model with complex network theory to investigate the volatility spillover network in the G20 stock markets. They find that volatility originating in one market rapidly spreads through the network, with the largest volatility receiver and sender being South Korea and Brazil. Weiping et al. (2020) extend this study and divide the G20 stock markets into four different spillover blocks. They highlight the effect of higher tariffs (mid-2018) imposed by the US government on other G20 markets and its economic impact. Both studies note that the interconnectedness of markets peaks during the global financial crisis in 2008, while evidence from the COVID-19 period is not included in the sample period. A further drawback of these studies is that capital is mobile not only across borders but also asset types. This means that in addition to the G20 markets, other well-established global assets cannot be ignored. For example, this could include evidence of interdependence between stocks and assets, including, oil, gold, and US 10-year Treasury bonds as key indicators of global risk.

To address the identified gaps in the literature, this paper incorporates key global non-stock assets (oil, gold, and bonds) into the G20 market network and extends the analysis to include the COVID-19 period. This allows for a wider perspective including the effect of assets that are considered as both safer in comparison to stocks (e.g. gold and bonds) as well as alternative risky ones (e.g. oil), including across a period of heightened global risk.

The aims of this paper are twofold. First, to compare the network nature of spillovers across the GFC and CVC periods. This will allow a better understanding of the impact of both crises, where no such analysis currently exists. Second, to consider the source of volatility spillovers between G20 stock markets and key global financial assets over different periods.

The main findings and their economic implications can be noted as follows. Volatility spillovers between global financial indicators and G20 stock markets in all sub-periods are significant and exhibit time-variation, with a high level of market interrelation. Importantly, volatility spillover networks are denser during the crisis periods of the GFC and CVC compared to non-crisis period networks. The implication of this finding demonstrates that crisis periods are more transitive in terms of volatility spillovers, which causes volatility to spread more rapidly through major financial indicators to G20 stock markets. These results should be of interest to investors seeking to diversify their portfolios across both asset types, including oil, gold, and bonds, and G20 stock markets. Moreover, the nature of diversification depends on both time and market specific information. Notably, during crisis periods, correlations between all markets increase and diversification opportunities become more restricted.

## Empirical methodology

The first step in the methodology is to extract the volatility spillovers and second, to construct the network and associated statistics.

### Spillover extraction and construction

*Step 1: Synchronize pairwise closing prices and calculate the log return series*

The trading days across the G20 stock markets differ between market pairs. Therefore, we need to find the intersection between market closing prices. To obtain the common trading days for any two markets, we synchronize the pairwise closing prices for which both markets have active trading. Having conducted the pairwise synchronization, logarithmic returns are calculated by the following Eq. (1):

1 $${R}_{i,t}=[\mathrm{ln}\left({P}_{i,t}\right)- \mathrm{ln}\left({P}_{i,t-1}\right)]*100$$

where ${P}_{i,t}$ is the closing price of stock index for country $i$ at time $t$ and ${P}_{i,t-1}$ is the closing stock price index for country $i$ at time $t-1$.^1^

*Step 2: Employ the bivariate GARCH-BEKK model to extract volatility spillovers*

To capture volatility spillovers between pairwise markets, we apply the GARCH-BEKK model of Engle and Kroner (1995). In considering multivariate-GARCH models there is a range of alternatives including the CCC (constant conditional correlation; Bollerslev 1990), DCC (dynamic conditional correlation; Engle 2002) and the GARCH-VECH (Bollerslev et al. 1988) models in addition to the GARCH-BEKK. A key advantage of the GARCH-BEKK model is that it does not impose any restriction on the conditional correlation structure between series. In addition, the conditional variances are restricted to ensure they are positive definite, while reducing parameter dimensions. In contrast, the CCC and DCC models do not capture spillover effects from one market to another, while the GARCH-VECH requires a large number of parameters (23) to be estimated and this can often lead to non-convergence. Furthermore, the restricted form of the GARCH-VECH, known as the diagonal GARCH-VECH model, may have better convergence properties but it does not generate cross-product (spillovers) parameters. As the GARCH-BEKK does not suffer from these issues and is used within the cogent literature (see, An et al. 2020; Liu et al. 2017; Weiping et al. 2020; Zhang et al. 2020a, b), we conclude that the GARCH-BEKK model provides the preferred estimation approach. We use a single lag for the sake of parsimony.

For the GARCH-BEKK model, the mean equation is given as:

2 $$R\left(t\right)=\left[ \begin{array}{c}{R}_{1}(t)\\ {R}_{2}(t)\end{array} \right]= \left[ \begin{array}{c}{\mu }_{1}(t)\\ {\mu }_{2}(t)\end{array} \right]+\left[ \begin{array}{cc}{\varphi }_{11} & {\varphi }_{12}\\ {\varphi }_{21}& {\varphi }_{22}\end{array} \right] \left[\begin{array}{c}{R}_{1}(t-1)\\ {R}_{2}(t-1)\end{array}\right]+ \left[ \begin{array}{c}{\varepsilon }_{1}(t)\\ {\varepsilon }_{2}(t)\end{array} \right]$$

where ${R}_{t}$ is the logarithmic return that is a ($2\times 1)$ vector of market 1 and market 2 at time *t*, ${\mu }_{1}(t)$ and ${\mu }_{2}(t)$ represent the long-term drift coefficient, and then ${\varepsilon }_{1}(t)$ and ${\varepsilon }_{2}(t)$ are the random errors at time *t*. The variance–covariance equation is then given by:

3 $$H\left(t\right)= {C}^{{\prime}}C+{A}^{{\prime}}{\varepsilon }_{t-1}{\varepsilon }_{t-1}^{{\prime}}A+{B}^{{\prime}}{H}_{t-1}B$$

4 $$C= \left[ \begin{array}{cc}{c}_{11} & 0\\ {c}_{21}& {c}_{22}\end{array} \right], A= \left[ \begin{array}{cc}{a}_{11} & {a}_{12}\\ {a}_{21}& {a}_{22}\end{array} \right] and B= \left[ \begin{array}{cc}{b}_{11} & {b}_{12}\\ {b}_{21}& {b}_{22}\end{array}\right]$$

where $H\left(t\right)$ is the conditional covariance matrix ($2\times 2)$. $C$ is the constant coefficient terms in the form of a lower triangular matrix. The coefficient matrices of the GARCH-BEKK model are given by $A$ and $B$. $A$ represents the parameters of the conditional residual matrix, and $B$ is the conditional covariance matrix’s parameters. The diagonal elements of A and B such as ${a}_{11}, {a}_{22}, {b}_{11}, and {b}_{22}$ measure their own markets’ previous shocks (ARCH effect) and volatility (GARCH effect), while the off-diagonal parameters in the matrices (${a}_{12}, {a}_{21}, {b}_{12}, and {b}_{21}$) quantify the cross-stock market effects of shocks and volatility between stock markets $i$ and $j$, in other words, the volatility spillovers. The bivariate GARCH-BEKK model is estimated with 17 parameters in total, which does not impose any restriction on the conditional correlation structure between the model variables.

The GARCH-BEKK model is estimated using the maximum likelihood method, with the conditional log likelihood function written as.

5 $$L\left(\theta \right)=-T \mathrm{ln}\left(2\pi \right)-\frac{1}{2}\sum_{t=1}^{T}[ \mathrm{ln}\left|{H}_{t}\left(\theta \right)\right|+{\varepsilon }_{t}{\left(\theta \right)}^{{\prime}}{H}_{t}^{-1}{\varepsilon }_{t}\left(\theta \right) ]$$

where $T$ is the number of observations, and $\theta$ is the vector of parameters to be estimated. The influence of volatility spillover from stock market $i$ to stock market $j$ is calculated as the absolute sum of the off-diagonal values of matrices $A$ and $B$, which can be defined as follows:

6 $${e}_{\mathrm{1,2}}=\left|{a}_{12}\right|+\left|{b}_{12}\right|$$

where ${a}_{12}$ and ${b}_{12}$ represent the off-diagonal elements of matrices $A$ and $B$, respectively. ${e}_{\mathrm{1,2}}$ stands for the magnitude of volatility spillover effect from market 1 to market 2. Similarly, ${e}_{\mathrm{2,1}}$ is the size of volatility spillover from market 2 to market 1. ${e}_{1,2}+{e}_{\mathrm{2,1}}$ is the total size of bidirectional volatility spillover relation between markets 1 and 2.

*STEP 3: Construct the spillover network considering complex network construction rules*

Complex network theory takes into account the relations among different parts of a real complex financial system as a network (Hao et al. 2015; An et al. 2014). A complex network is a collection of nodes that are connected by edges. A complex network system is symbolised in Eq. (7):

7 $${\text{G}} \, {=} \, ({\text{N}},\, {\text{E}})$$

where *G* represents a complex network. *N* refers to the set of nodes and *E* is the set of edges between nodes. In the context of this study, our complex networks have some characteristic features such as the small world effect and the superposition phenomenon. The small world effect (Watts and Strogatz 1998) is a phenomenon in network theory that no node is independent from the network, which means that all nodes are linked to each other either with a direct or indirect tie. Two widely used network statistics, which are the average shortest path length and average clustering coefficient are used to determine the small world effect. Another network characteristic is the superposition phenomenon, which is a principle in physics applying all the linear systems such as height in a water wave, intensity of a light wave or pressure in a sound wave. For example, where two water waves are travelling in opposite directions, the size of combined wave is the sum of both water waves at the intersection point. Similarly, the thickness of an edge between pair nodes is identified by the superposition principle in this work.

To build a complex network, we prepare the nodes (as data frame) and edges (as matrix element) in the form of a square matrix whose main diagonal consists of zeros. Thus, the square matrix of spillover relation for the G20 stock markets can be shown by Eq. (8):

8 $$M=\left[\begin{array}{ccc}{e}_{\mathrm{1,1}}& \cdots & {e}_{1,\mathrm{n}}\\ \vdots & \ddots & \vdots \\ {e}_{\mathrm{n},1}& \cdots & {e}_{\mathrm{n},\mathrm{n}}\end{array}\right]$$

where *M* is the matrix of edges, which creates the complex network. Here, we consider the G20 stock markets and other global assets as the nodes. In a similar vein, the volatility spillovers ($e$) are considered as unidirectional or bidirectional edges between the nodes. Using the off-diagonal elements of the GARCH-BEKK model, we create a complex network of the global financial system. If the estimated off-diagonal parameters of the GARCH-BEKK are significant, it means that there is a volatility spillover effect from market 1 to market 2 and then the spillover value (${e}_{\mathrm{1,2}}$) from market 1 to market 2 is entered into the specified cell of matrix.^2^ The direction of spillover effect is shown with an arrow mark from market 1 to market 2. If there is also reverse spillover relation (market 2 to market 1), the spillover is bidirectional and arrow marks appear on both edges. In identifying the spillover relations, previous studies (Liu et al. 2017; Weiping et al. 2020; Zhang et al. 2020a, b) use the 10% significance level. They argue that this is because a too strict significance level may miss important spillover relations.

### Spillover network statistics

#### Weighted in-degree and weighted out-degree

The weighted degree of the spillover network indicates how strong the effects of the volatility spillovers are. If a node has a larger weighted degree, it has more potential to affect or be affected, depending on the weighted in/out degree, where the weighted-in-degree receives spillovers and the weighted-out-degree causes spillovers. The weighted in and out degrees are expressed by Eqs. (9) and (10) as follows:

9 $${w}_{i}^{\mathrm{in}}=\sum_{j=1}^{m}{e}_{ij}$$

10 $${w}_{i}^{\mathrm{out}}=\sum_{j=1}^{n}{e}_{ji}$$

where ${w}_{i}^{\mathrm{in}}$ and ${w}_{i}^{\mathrm{out}}$ represent the weighted in-degree and weighted out-degree, respectively. ${e}_{ij}$ implies the size of volatility spillover from node $i$ to node $j,$ and ${e}_{ji}$ implies the amount of volatility spillover from node $j$ to node $i$. $e$ is obtained by Eq. (6). $m$ and $n$ denote the number of edges that node $i$ has with the other nodes of network. A higher weighted degree means stronger volatility spillover relation in a complex network.

#### Average shortest path length and network diameter

In a complex network, the small world phenomenon (Watts and Strogatz 1998) means that all nodes are linked either with a direct or indirect tie. For detecting this phenomenon Watts and Strogatz (1998) suggest looking at both the average shortest path length and network diameter statistics. The average shortest path length is defined as the mean of the shortest steps of volatility spillover propagation from node $i$ to node $j$. It is expressed by Eq. (11):

11 $$r = \mathop \sum \limits_{i,j} \frac{{d_{ij} }}{{n\left( {n - 1} \right)}} {;} \quad \left( {i \ne j} \right)$$

where $r$ denotes the average shortest path length; the smaller $r$ in a network, the more linkages between nodes, which indicates a denser network (a larger $r$ denotes less linkages between nodes and a looser network). ${d}_{ij}$ is the shortest distance from node $i$ to node $j$. The denominator, $n\left(n-1\right)$, shows the maximum number of possible edges in the spillover network where $n$ is the total number of nodes. The network diameter is ‘the shortest path between the two most distant nodes of the network’. Once the shortest path length of every single node with respect to the other nodes is calculated, the network diameter is the longest one among all the calculated shortest path lengths. A smaller or larger value of diameter is interpreted same as the shortest path length.

#### Graph density and average clustering coefficient

The graph density indicates how close the number of edges is to the maximum number of possible edges in the network. If the graph density is equal to 1, the network is called a complete graph and includes all the possible edges. The network graph density can be calculated by Eq. (12) as follows:

12 $$D=\frac{2|E|}{|n|(\left|n\right|-1)}$$

where *E* is the number of edges between the nodes of network and $n$ is the total number of nodes. This measure amounts to a ratio of actual connections to potential connections. Another network statistic is the average clustering coefficient, which is a similar measure and shows how the nodes are integrated in the network graph. It is calculated by dividing the number of edges connecting a node’s neighbours to the total possible number of edges between the node’s neighbours.

## Data

The G20 is an international forum, consisting of 19 major developed and emerging countries (with the EU as a whole also represented). From a global perspective, the G20 accounts for 85% of Gross World Product and 80% of world trading (2014) and thus, represents an important bloc. However, capital is mobile across asset types, such that other (global) assets should be considered alongside stocks. Therefore, in examining spillovers, we also include oil, bonds and gold market information.

Specifically, we employ the daily closing prices of 19 major stock market indices of the G20 countries; which are S&P MERVAL (Argentina), S&P ASX200 (Australia), BOVESPA (Brazil), GSPTSE (Canada), SHCI (China), CAC40 (France), DAX30 (Germany), SENSEX (India), IDX (Indonesia), Italy 40 (Italy), NIKKEI225 (Japan), KOSPI (Korea), S&P BMV (Mexico), MOEX (Russia), TADAWUL (Saudi Arabia), SOUTH AFRICA TOP40 (South Africa), BIST100 (Turkey), FTSE100 (UK), and SP500 (US).^3^ Additionally, crude oil (WTI; West Texas Intermediate), gold returns and the US 10-year Treasury bond yields are included. All the data are extracted from the ‘investing.com’ website, at the daily frequency and over the sample period between January 8, 2003 and January 4, 2021, which includes both periods of calm and turmoil.

Table 1 presents the descriptive statistics of all series used in this study after synchronising the data as noted above, this leads to approximately 4400 observations for each series. The summary statistics are as expected, with a mean daily return that is close to zero and a larger standard deviation. The skewness and excess kurtosis statistics show that the return series are leptokurtic with higher peak points as well as fatter tails. The Jarque–Bera normality test results indicate that the distribution of each series is not Gaussian. The Augmented Dickey-Fuller (ADF) test results reject the null hypothesis of a unit root at the 1% significance level, with all series stationary. Serial correlation tests for both the mean (Ljung-Box Q-statistic) and variance (ARCH-LM test) indicate the presence of such correlation, which supports the use of the models outlined in Eqs. (2)–(4).

**Table 1:** Summary statistics

	Mean	SD	Skew.	Ex. Kurt.	JB Stat.	ADF	Q(20)	ARCH(1)
Argentina	0.106	2.309	− 1.398***	21.537***	82,701.4***	− 23.549***	41.597***	21.453***
Australia	0.016	1.061	− 0.721***	8.408***	13,811.1***	− 48.044***	49.558***	577.262***
Brazil	0.054	1.777	− 0.376***	7.705***	10,686.1***	− 14.432***	58.796***	540.84***
Canada	0.020	1.107	− 1.123***	21.120***	84,301***	− 12.453***	148.653***	757.594***
China	0.031	1.947	− 0.379***	5.954***	4555.17***	− 7.823***	53.765***	53.125***
France	0.012	1.394	− 0.169***	8.958***	15,022.3***	− 24.047***	49.609***	222.499***
Germany	0.033	1.401	− 0.233***	7.260***	9885.87***	− 67.336***	25.465	85.511***
India	0.061	1.454	− 0.264***	10.454***	19,474.8***	− 17.628***	83.539***	211.144***
Indonesia	0.063	1.334	− 0.530***	7.308***	9636.64***	− 59.179***	63.849***	206.137***
Italy	0.042	0.748	− 0.605***	1.735***	177.9***	− 33.096***	14.030*	9.372***
Japan	0.028	1.508	− 0.202***	10.531***	19,474.3***	− 23.729***	52.068***	230.224***
Korea	0.035	1.303	− 0.455***	7.432***	10,161.6***	− 12.961***	45.727***	338.596***
Mexico	0.044	1.227	− 0.204***	7.157***	9317.0***	− 15.878***	63.192***	150.833***
Russia	0.054	1.950	− 0.314***	20.147***	73,640.3***	− 10.338***	104.100***	73.716***
S. Arabia	0.039	1.895	− 1.196***	13.869***	25,038.7***	− 14.263***	71.399***	129.999***
S. Africa	0.043	1.358	− 0.162***	5.692***	5744.59***	− 15.203***	47.441***	257.982***
Turkey	0.059	1.675	− 0.314***	3.997***	2972.02***	− 28.556***	38.799***	59.164***
UK	0.010	1.163	− 0.357***	9.814***	18,034.5***	− 13.503***	67.922***	216.446***
US	0.031	1.225	− 0.547***	13.831***	35,853.9***	− 14.511***	207.461***	501.845***
WTI (oil)	0.015	2.644	− 0.172***	16.065***	47,132.9***	− 11.322***	690.461***	1059.56***
10Y bond	− 0.032	2.616	0.010***	31.433***	187,479***	− 11.171***	153.38***	1223.85***
Gold	0.053	1.717	− 0.169***	11.603***	18,043.4***	− 38.333***	117.108***	99.894***

Asterisk *, **, and *** denote rejections of null hypothesis at 10%, 5%, and 1% significance levels, respectively. The null hypothesis of the third and fourth moments are “Skewness = 0” and “Excess Kurtosis = 3”

## Empirical results

This paper seeks to examine volatility spillovers between key global financial assets and G20 stock markets, which compares with the current literature that typically analyses a small selection of stock markets or one stock market with alternative assets. To study this relation, we use a synthesis first developed by Liu et al. (2017), which combines the bivariate GARCH-BEKK model with the complex network theory.^4^ To examine this spillover network of international markets we use daily data over the period 08/01/2003–04/01/2021, which is divided into five sub-periods that cover tranquil and crisis periods. Two important crisis periods within our sample are the Global Financial Crisis (GFC) in 2008 and the COVID-19 Crisis (CVC) in 2020, and these act as the cornerstone of this study.

Specifically, we divide our full sample into sub-periods in accordance with the crisis and non-crisis periods. Period 1 captures the Pre-Crisis period and is from January 8, 2003 to August 9, 2007. Period 2 encompasses the 2008 Global Financial Crisis, covering August 10, 2007 to December 30, 2009. Period 3 (Post-Crisis) takes place between January 4, 2010 and December 16, 2013. Period 4 is the Pre-Pandemic period and covers December 17, 2013 to December 30, 2019. Period 5 is from January 4, 2020 to January 4, 2021, and is the COVID-19 Crisis period.

In choosing these dates, we follow the work of Weiping et al. (2020) and Zhang et al. (2020a, b) who also use similar sub-sample analysis. Our sub-sample dates differ slightly from these two papers as we use a larger sample, both starting earlier (in 2003 compared to 2006) and ending later (in 2021 compared to 2018). The end of our Period 1 and the dates for Period 2 are the same as these papers, while our Period 3 matches that of Weiting et al. (2020). We extend our Period 4 beyond the sample in each paper, and this allows us to isolate the COVID-19 period. While the choice of sub-sample dates always contains an element of subjectivity, by following the previous literature, we are able to provide some comparability.^5^

Table 2 presents some widely used networks statistics. The first statistic, column ($\mathrm{i}$), shows that there are 171, 257, 142, 175, and 297 volatility spillover linkages that are extracted from the 462 edges in the 5 sub-periods, respectively. In column ($\mathrm{ii}$), the total degree indicates the sum of all node sizes in the sub-periods and whose values are highly correlated with the values in column ($\mathrm{i}$). These measures are two of the more important general network statistics and indicate that volatility spillover between global financial indicators and G20 stock markets are present in all sub-periods and that the spillover relations are largely bidirectional. More importantly, volatility spillover networks are more dense during a crisis, as evidenced by the values in Period 2 (GFC in 2008) and Period 5 (CVC in 2020) when compared to the non-crisis Periods 1, 2, and 4. It is also clear that the volatility spillover network is time-varying with the number of linkages changing over the five sub-periods.

**Table 2:** Network statistics

	Number of edges ($i$)	Total degree ($ii$)	Av. weighted degree ($iii$)	Av. shortest Path length ($iv$)	Network diameter ($v$)	Graph density ($\nu i$)	Av. clustering coefficient ($\nu ii$)
Period 1	171	24.95	1.134	1.463	3	0.541	0.615
Period 2	257	72.28	3.286	1.329	2	0.671	0.699
Period 3	142	36.02	1.638	1.602	3	0.420	0.539
Period 4	175	29.81	1.355	1.424	2	0.576	0.608
Period 5	297	206.9	9.406	1.225	2	0.775	0.808

Smaller the values of ($\mathrm{iv}$) average shortest path length and ($\mathrm{v}$) network diameter mean tighter networks. The others are meant to be as normal; more precisely, higher values, tighter networks are

Of the other statistics, in column ($\mathrm{iii}$), the average weighted degree is the ratio of total degree to the number of nodes. The higher the average weighted degree, the tighter the market interrelations. These values are higher in the crisis periods 2 and 5 (3.286 for GFC and 9.406 for CVC) compared to the non-crisis periods 1, 3, and 4 (1.134, 1.638, and 1.355, respectively). The implication is that crisis periods are likely to deepen market connections and therefore, the potential for financial contagion. Fluctuations occurring in one market can therefore, spread more easily to other markets. For column ($\mathrm{iv}$), the average shortest path length is defined in a network as the mean shortest steps of volatility spillover propagation from node $i$ to node $j$. A smaller shortest path length means stronger linkages between nodes, which also mean a denser network. This statistic fluctuates between the values 1 and 2, with lower values in the crisis periods (1.329 and 1.225 for periods 2 and 5, respectively) compared to the networks of non-crisis periods (1.463, 1.602, 1.424 for periods 1, 2 and 4, respectively). Hence, volatility spillovers in crisis periods propagate faster compared to those in non-crisis periods.

In column ($\mathrm{v}$) the network diameter is known as the shortest path between the two most distant nodes of the network. The network diameter is at 2 for the second, fourth, and fifth sub-periods, while the same statistic is at 3 for the first and third periods. This means that any spillover in any node may reach the farthest node point in maximum 2 steps in the crisis periods, whilst it is a maximum of 3 steps in normal periods. Again, this is the evidence of stronger spillover transmission during the crisis periods. In column ($\mathrm{\nu i}$), the graph density is a measure that shows how close the number of edges is to the maximum possible number of edges. If the graph density is equal to 1 in a network, the network is called a complete graph that includes all the possible edges. The graph density statistics are closer to 1 in the turmoil periods 2 and 5 (0.671 and 0.775, respectively) in comparison with the calm periods 1, 3, and 4 (0.541, 0.420, and 0.576, respectively). Column ($\mathrm{\nu ii}$), the average clustering coefficient, is a similar measure to graph density, indicating how the nodes are integrated in a network graph. The graph density and average clustering coefficient move together to a large extent; the two peak points of the average clustering coefficient are approximately 0.7 and 0.8 in the GFC and CVC periods, respectively.

In sum, the network statistics present three key results. First, spillover effects exist through the five different sub-periods. Second, the nature of such spillovers is time-varying across the sample periods. Third, the strength and nature of spillovers increase during a crisis.

### Results by period

#### Crisis periods 2 and 5

**Fig. 1:**
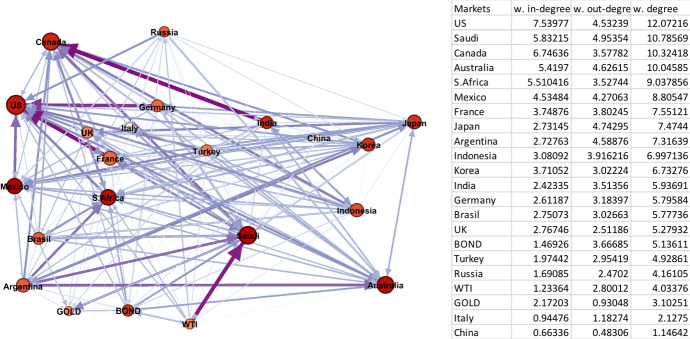
Period 2 (10/08/2007–30/12/2009 Global Financial Crisis). Note: Darker colours in networks represent larger spillover relationships, while lighter colours indicate weaker spillover relationships; red and bigger nodes show bigger spillover centres, wider and dark purple edges are the strongest linkages. The table on the right hand side is sorted by the average weighted degree values of the markets from largest to smallest. (Color figure online)

**Fig. 2:**
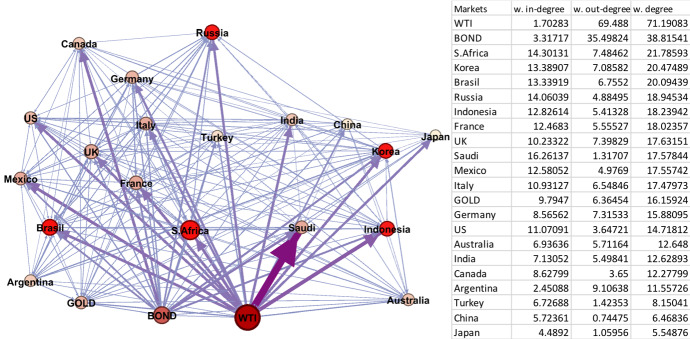
Period 5 (04/01/2020–04/01/2021 Covid Crisis). Note: Darker colours in networks represent larger spillover relationships, while lighter colours indicate weaker spillover relationships; red and bigger nodes show bigger spillover centres, wider and dark purple edges are the strongest linkages. The table on the right hand side is sorted by the average weighted degree values of the markets from largest to smallest. (Color figure online)

The crisis periods 2 and 5 cover the GFC and CVC and thus, it is of interest to see the different impact of the two crises. Notably, we can observe a clear difference in the network graphs, presented in Figs. 1 and 2, and statistics noted in Table 2 for the two periods. Comparing the two figures, the spillover network graph in Period 5 is tighter and more integrated than the network graph of Period 2. This suggests that volatility spillovers among markets in the CVC period spreads faster than volatility spillovers in the GFC period. This implies that the CVC period exhibits more widespread effects on both key global assets and the G20 markets compared to the GFC period in 2008. This can equally be seen in the number of edges (297 in period 5 against 257 in period 2), the total degree (207 against 72), average weighted degree (9 against 3) and graph density (0.8 against 0.7).

In Period 2 (Fig. 1), the rankings of top weighted nodes are USA, Saudi Arabia, Canada, and Australia, respectively, while the stock markets of China and Italy are the bottom weighted. More specifically, USA, Canada, Saudi Arabia, and Australia have the highest weighted-in-degree that receive spillovers, while Saudi Arabia, Japan, Australia, Argentina, and USA are the top weighted-out-degree volatility senders, respectively. Thus, we can see similarity in the markets that are the highest volatility receivers (weighted-in-degree) and senders (weighted-out-degree). For example, the USA appears as the most active node with the largest weighted degree.

While the GFC began in the USA, we can observe the effect spreading to the rest of the world through interconnections in the global financial system. We can see that Canada and Australia, which rely on commodity exports are notably affected. Equally, a further market affected is the major oil-exporting Saudi Arabia, while South Africa is also a commodity exporter. We can also see the role played by the global risk factors of interest rates, oil, and gold prices play in the transmission. Although, they appear towards the bottom of the weighted list, they play a more significant impact than several G20 markets.

These results imply the existence of systematic risk on a global scale that can be thought of as restricting the possibility of international portfolio diversification. However, a small number of markets during this period, such as China and Italy, could be used to construct more robust diversified portfolios as the node sizes of these markets are the smallest in the network.

In Period 5 (Fig. 2), the average weighted degrees are much higher compared to their counterparts in Period 2. A notable difference is that no specific stock market can be regarded as the source of the crisis compared to Period 2 and the US market. In Period 5, the oil and bond markets are shown to be the source of the largest amount of volatility spillovers directed to the G20 markets. In terms of the G20 stock markets themselves, the largest weighted degrees are South Africa, Korea, and Brazil, whereas Japan, China, and Turkey have the smallest weighted degree values, respectively. Looking deeper into the Period 5 results across the weighted degrees, the top volatility senders (out-degree) are different from the receivers (in-degree). As noted, the global financial indicators of oil and bonds are the volatility senders, meaning that they affect but are not affected by other markets. The top receivers are the stock markets of Saudi Arabia, South Africa, Russia, Korea, and Brazil, respectively. This again, notably, highlights the importance of oil within global financial markets, with major oil export markets affected.

In explaining the large volatility spillovers from oil prices to the G20 markets, we saw reduced global oil demand by 29 million barrels per day during the COVID the lockdown period, while the price of West Texas Intermediate (WTI) fell to negative $37 per barrel on April 20, 2020. This oil price crash creates a large spike on own volatility that spread to oil dependent markets as well as globally. Following oil, the node on US 10-year Treasury bond yields is the second largest that has an impact on G20 markets. The bond yields start decreasing when Wuhan lockdown began and reach the lowest level (below 0.6) with the declaration of coronavirus pandemic by the World Health Organisation. In contrast, the spillover effect from gold prices on the G20 stock markets is relatively smaller.

#### Non-crisis periods 1, 3, and 4

**Fig. 3:**
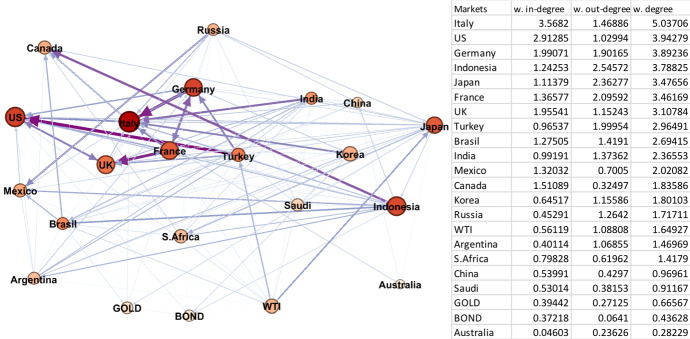
Period 1 (Pre-Crisis 08/01/2003–09/08/2007). Note: Darker colours in networks represent larger spillover relationships, while lighter colours indicate weaker spillover relationships; red and bigger nodes show bigger spillover centres, wider and dark purple edges are the strongest linkages. The table on the right hand side is sorted by the average weighted degree values of the markets from largest to smallest. (Color figure online)

**Fig. 4:**
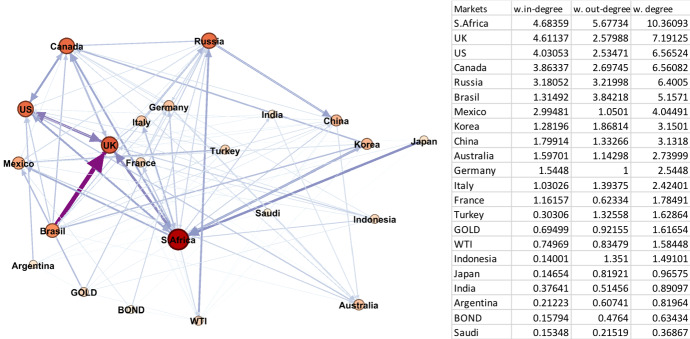
Period 3 (Post-Crisis 04/01/2010–16/12/2013). Note: Darker colours in networks represent larger spillover relationships, while lighter colours indicate weaker spillover relationships; red and bigger nodes show bigger spillover centres, wider and dark purple edges are the strongest linkages. The table on the right hand side is sorted by the average weighted degree values of the markets from largest to smallest. (Color figure online)

**Fig. 5:**
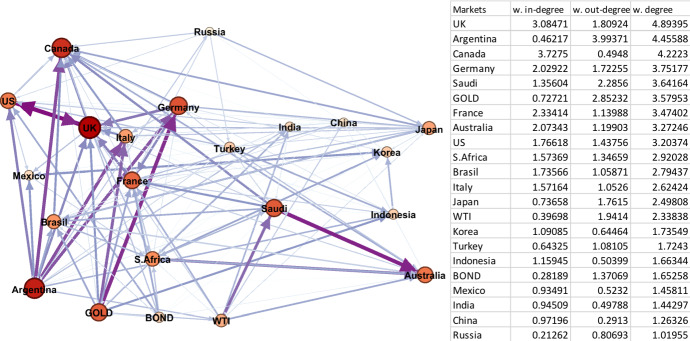
Period 4 (Pre-Pandemic 17/12/2013–30/12/2019). Note: Darker colours in networks represent larger spillover relationships, while lighter colours indicate weaker spillover relationships; red and bigger nodes show bigger spillover centres, wider and dark purple edges are the strongest linkages. The table on the right hand side is sorted by the average weighted degree values of the markets from largest to smallest. (Color figure online)

The network graphs (Figs. 3, 4 and 5) of the non-crisis periods are less dense than for the crisis periods. Furthermore, unlike the crisis periods, the influence of the global assets (oil, gold, and bond) on the G20 markets is limited.

In Period 1 (Fig. 3), the node in Italy has the largest weighted degree among markets, while its value in Periods 3 and 4 is relatively smaller. This emphasises the time-varying nature of the volatility spillover relations among the G20 markets. The most active nodes in Period 1 following Italy are from the USA, Germany, Indonesia, Japan, and France. As a most active node, Italy is the largest volatility receiver, but not the largest volatility sender. The ranking of the largest senders is Indonesia, Japan, and France. The smallest nodes during this first period are Australia, bonds, gold, Saudi Arabia, and China. In Period 1, the impact of oil, bonds, and gold on the G20 stock markets is notably limited.

In Period 3 (Fig. 4), the ordering of markets changes reflecting the time-variation in spillovers. Here, the most active nodes are South Africa, UK, USA, and Canada, while Saudi Arabia, bonds, Argentina, India, and Japan are the least active ones. Notably, South Africa, Brazil, Russia, UK, and USA are the largest volatility senders, while South Africa, UK, USA, and Canada are the largest volatility receivers.

In Period 4 (Fig. 5), UK, Argentina, Canada, and Germany exhibit the largest set of nodes, whereas Russia and China have the smallest. The biggest volatility senders are Argentina, Saudi Arabia, gold and the UK, with Canada, UK, and France the biggest volatility receivers. In terms of the global financial indicators, gold is a significant volatility sender that affects the G20 markets, but oil and bonds have a moderate effect. Of note, the price of gold saw a gradual increase towards the end of the period, just before the beginning of the CVC. This time period includes the Brexit vote and this may explain the high degree of UK spillovers.

### Key paths in spillover networks

The spillover relations are conveyed by the edges among the nodes in a complex network and therefore detecting the thickest linkages between markets may reveal some important information about market interrelations. Table 3 presents the top five edges (strongest linkages) over each of the sub-periods. It is worth noting that the top edges are independent from the size of nodes, meaning that a node could be relatively small, while having the top edge of network. For instance, the node of India is relatively small in Period 2, but it has the largest linkage in the same period, which is from India to Canada.

**Table 3:** Key paths in spillover networks

	Value (%)		Value (%)		Value (%)
**Period 1**		**Period 2**		**Period 3**	
Turkey –> USA	0.71 2.8%	India –> Canada	1.02 1.4%	Brazil –> UK	1.55 4.3%
Germany –> Italy	0.66 2.6%	France –> US	1.00 1.3%	UK –> USA	0.93 2.5%
France –> UK	0.64 2.5%	OIL –> Saudi	0.96 1.3%	US A –> UK	0.90 2.4%
Indonesia–> Canada	0.51 2.0%	Germany –> USA	0.87 1.2%	S. Africa –> UK	0.86 2.3%
France –> Germany	0.50 2.0%	Argentina –> Australia	0.69 0.9%	Japan –> S. Africa	0.80 2.2%
**Period 4**		**Period 5**			
UK –> US	0.70 2.3%	OIL –> Saudi	11.3 5.4%		
Saudi –> Australia	0.65 2.1%	OIL –> Indonesia	5.63 2.7%		
US –> UK	0.65 2.1%	OIL –> Mexico	4.37 2.1%		
GOLD –> Germany	0.62 2.0%	OIL –> S. Africa	4.12 1.9%		
Argentina –> Canada	0.54 1.8%	BOND –> Canada	4.02 1.9%		

“–>” indicates the spillover direction, the first number (value) is the amount of volatility the second (per cent) is the percentage of spillover amount in total spillover

Examining the results in Table 3, Period 5 has the strongest linkages among all the sub-periods, with spillovers from oil to Saudi Arabia, Indonesia, Mexico, and South Africa. The strongest edge is from oil to Saudi Arabia with a 5.4% weight of the total spillover. This highlights the importance of oil to the Saudi stock market given its major oil-exporting role. As noted above, both oil demand and the oil price fell dramatically at the start of the CVC period. Mexico and Indonesia are also notable for oil exports. In Period 2, the direction of top edges is towards the node of US market. This means that despite the US being the origin of the GFC crisis, the US market is also exposed to significant external volatility, with the top two edges from France and Germany. Again, Saudi Arabia is notably affected as the GFC led to a global recession and a fall in demand for oil. This provides further evidence of a strong relation between oil prices and the Saudi stock market.

In Period 3, the top edge is from Brazil to the UK, while other significant edges take place bidirectionally between the UK and US markets. Japan to South Africa and South Africa to the UK also show notable linkages. The bidirectional spillover relation between the US and UK stock markets returns in Period 4. It is followed by edges from Saudi Arabia to Australia, Gold to Germany, and Argentina to Canada. In Period 4, some economic implications regarding the largest edge between the UK and US markets can be linked to the Brexit referendum (23 June 2016) that has a significant impact on the UK economy and reveals the UK as large volatility source. Afterwards, the 2015–2016 stock market sell-off in the US market and the world-wide stock market downturn in 2018 can further explain the strong linkage between the UK and US stock markets. In Period 1, the largest linkages are more mixed, being from Turkey to the US, Germany to Italy, France to the UK, Indonesia to Canada, and France to Germany.

### Network robustness checks

Testing the robustness of our complex network can ensure its stability and resilience and the reliability of the presented results. To consider robustness in a complex network, we can examine the response of the network to changes in the number of nodes. Therefore, we re-examine each network by, first, adding a further node and, second, by removing the global financial indicators from our sample dataset. Inevitably, changing the number of nodes will alter the results, however, the key question is whether this results in substantial changes to the network statistics and node sizes. The results (available upon request) evidence that no significant changes to the network statistics and node-edge sizes of the included markets are observed across the sub-periods. This underpins the consistency of our results.

Specifically, we undertake two exercises and consider how the network statistics change with a differing number of nodes. First, we increase the number of nodes from 22 to 23 by including the implied volatility index, VIX, based on the US SP500. Here, the average weighted degree, graph density, and clustering coefficient for all sub-periods rise proportionally to indicate that the networks with 23 nodes are slightly tighter compared to those of 22. However, the average shortest path length and network diameter statistics, which are inversely proportional to the other three statistics, decrease slightly. In the second exercise, the number of nodes decreases from 22 to 19 by removing the three global financial indicators. Here, we do not encounter any substantial changes in the network statistics (except for the average weighted degree in Period 5 that falls from 9.406 to 4.407).^6^ For the graph density and clustering coefficient, we see a small increase in the first four sub-periods, and a slight decrease in Period 5. Accordingly, the average shortest path length decreases in the first four sub-periods and increasing in the fifth period. The network diameter does not change in general, but the weighted degree results suggest an increase in the first and third periods and a decrease in other periods. To sum, these results do not affect our main conclusions as no significant changes in the node-edge sizes of networks are reported.

## Summary and conclusion

This paper analyses the volatility spillover relations between key global financial barometers (oil, gold, and bond) and G20 stock markets using a network approach. Specifically, a bivariate GARCH-BEKK model that captures spillover relations is combined with a complex network approach. Using this synthesis, we construct the spillover networks of international financial markets between 08/01/2003 and 04/01/2021 and divided this sample into five sub-periods to cover calm and crisis periods including the Global Financial Crisis (GFC) in 2008 and the Covid-19 Crisis (CVC) in 2020.

We detect 171, 257, 142, 175, and 297 volatility spillover linkages across the five sub-periods, respectively. This highlights the time-varying nature of the spillovers between the key global variables and G20 markets. Of note, the volatility spillover networks are much denser during the GFC and CVC crisis periods compared to the networks of non-crisis periods. The crisis periods are more transitive, resulting in volatility that transmits more rapidly and directly through the different assets examined. In Period 5 (CVC), the global financial indicators of oil and bonds appear as the main senders of volatility, indicating that they affect other markets but are not affected by those markets. In contrast, Saudi Arabia, Russia, South Africa, and Brazil are major volatility receivers in this period. An important point here is the role that oil plays in affecting global stock markets and notably oil-exporting markets. In Period 2 (GFC), as the crisis began in the USA, it acts as a major source of volatility spillovers along with Australia, Canada, and Saudi Arabia. A further point of interest is that China is among the least sensitive market to external volatility across all the sub-periods. In the non-crisis periods, the influence of the global variables on the G20 is notably lower compared to the case of the crisis periods. Instead, the effect of regional information becomes dominant, such as the Brexit referendum in the UK.

Although the results of this work are time and market specific, the movements of key nodes and edges over time provide important information. For policymakers, investors, and market participants, considering spillover relations in G20 stock markets is important in being able to manage risks and portfolio diversifications. For example, the existence of global risk factors can be thought as a sign to restrict the possibilities of portfolio diversification, especially during crisis periods when the correlations among investment instruments are high. In the non-crisis periods, more diversified portfolios can be constructed depending on time and market specific information.

## Footnotes

1 An alternative approach to the modelling outlined in the section is to use a VAR for the whole network and follow the general method of Diebold and Yilmaz (2014) and, more specifically, the adapted approach of Demirer et al. (2018). However, to use a single VAR would mean matching all series simultaneously (rather than pairwise) and result in a significant loss of data (approx. 45% of original the data would be lost).

2 If the estimated parameters are insignificant, no spillover relation between pairwise markets and enter 0 into the specified matrix element.

3 In determining the choice of index, we follow those used by Weiping et al. (2020) and Zhang et al. (2020a, b) as well as being based on, arguably, the most recognisable index in each country.

4 To estimate the models, we use a range of software. Specifically, R studio (xts package) to match and prepare the pairwise data, Gretl (mgarch package) for GARCH-BEKK model and Gephi for networks construction.

5 An alternative approach, as considered by Demirer et al. (2018) is to estimate a rolling full-sample VAR. However, as noted above, this would mean a substantial loss of information in aligning the data.

6 This change in Period 5 is linked to the crash in oil prices (20 April 2020) that have a notable impact on the network. Therefore, when we omit the oil, bond, and gold in this exercise, we observe the noted fall.
